# Pediatricians’ Practice Patterns on the Revised 2022 Neonatal Hyperbilirubinemia Guidelines

**DOI:** 10.3390/pediatric18040101

**Published:** 2026-08-03

**Authors:** Estherline J. Thoby, Aimee Lariviere, Katherine Briski, Anna Petrova

**Affiliations:** 1 Department of Pediatrics, Rutgers Robert Wood Johnson Medical School, New Brunswick, NJ 08901, USApetroran@rwjms.rutgers.edu (A.P.); 2 Department of Pediatrics, Nemours Children’s Health, Wilmington, DE 19803, USA; katherine.briski@gmail.com

**Keywords:** jaundice, phototherapy, hyperbilirubinemia, neonatal

## Abstract

**Background/Objective:** In 2022, the American Academy of Pediatrics (AAP) revised the guidelines used to manage neonatal hyperbilirubinemia with a focus on reducing unnecessary testing and phototherapy. However, pediatricians’ knowledge and compliance with the current guidelines have not been assessed. We conducted a survey to evaluate New Jersey pediatricians’ current knowledge and practice patterns with the newly proposed guidelines. **Patients and Methods:** A questionnaire consisting of 28 closed-ended Likert-scale questions, along with demographic data, was distributed twice in 2024 to all members of the New Jersey AAP Chapter. Of 128 respondents, 120 who defined their involvement in the care of neonates with hyperbilirubinemia were analyzed. **Results:** The majority of survey respondents were general pediatricians (71.7%). Up to 70% recognized the risk factors for developing severe hyperbilirubinemia, except for Down Syndrome. Up to 60% of respondents utilized transcutaneous bilirubin in low-risk hyperbilirubinemia neonates and serum bilirubin after phototherapy initiation in high-risk neonates as recommended by the AAP. Almost all of the respondents followed the AAP recommended post-discharge follow-up and/or bilirubin measurement. The majority reported phototherapy initiation at the recommended thresholds; however, only 12.5% of surveyed pediatricians followed the recommended threshold for phototherapy discontinuation. **Conclusions:** Surveyed pediatricians in our study were most likely to comply with the 2022 AAP guidelines; however, opportunities remain for improving pediatricians’ awareness of specific risk factors, reducing unnecessary laboratory testing and discontinuation of phototherapy at the recommended level.

## 1. Introduction

Severe neonatal jaundice in healthy infants is increasingly recognized as a significant clinical concern in the 21st century [1,2]. This growing awareness arises from the potential risk of bilirubin-induced neurologic dysfunction (BIND), which affects approximately 2.4 infants out of every 100,000 live births in the United States [3]. Neonatal hyperbilirubinemia remains a critical issue that demands attention from pediatricians and neonatologists alike. In recent years, the risk of hyperbilirubinemia due to Rh incompatibility and exchange transfusion has significantly decreased due to the immunization of pregnant and postpartum women with anti-D immunoglobulin [4]. The American Academy of Pediatrics (AAP) has also taken significant steps to develop and promote comprehensive guidelines aimed at effectively managing hyperbilirubinemia in healthy newborns born at 35 weeks of gestation or later [5,6,7]. In 2022, the AAP published a revision of the neonatal hyperbilirubinemia guidelines [8]. Key updates of the revision included higher phototherapy thresholds based on gestational age and neurotoxicity risk factors, discontinuation of phototherapy when TSB has decreased by at least 2 mg/dL, obtaining rebound bilirubin at least 12 h after phototherapy discontinuation, and recommendations for discharge follow-up and post-discharge testing [8]. The 2022 updates from the AAP overall highlighted the importance of reducing phototherapy duration and bilirubin testing, empowering clinicians to minimize unnecessary interventions and optimize patient outcomes [8].

Since the implementation of the 2022 AAP guidelines, several studies have identified a decrease in the use of total serum bilirubin (TSB) testing and a reduced rate in the initiation of phototherapy [9,10,11]. Although there has been a decline in hospitalizations related to hyperbilirubinemia, this has not corresponded with a reduction in phototherapy use among low-risk infants, or differences in the rates of exchange transfusions or readmissions for phototherapy [11,12]. However, a survey conducted among pediatric hospitals in the US within the Academic Pediatric Association’s (APA) Better Outcomes through Research for Newborns (BORN) Network revealed significant variations in clinical practices and relatively low adherence to the 2022 AAP hyperbilirubinemia guidelines, particularly concerning newborns who were direct antiglobulin test (DAT) positive [13]. Inconsistency in management despite guideline updates may suggest a lack of consensus among pediatricians to align practices with the new recommendations.

Although a strong understanding of guidelines does not always correlate with effective clinical management, it is important to explore pediatricians’ knowledge and intent to follow the 2022 AAP recommendations in their practice. By identifying these areas, we can foster greater awareness and develop targeted educational strategies that support pediatricians in caring for infants with neonatal hyperbilirubinemia, ultimately enhancing patient care. Our survey was designed to evaluate pediatricians’ knowledge and intention to follow the revised guidelines. We conducted a survey of pediatricians to evaluate how well their current knowledge and intent to practice align with the new proposed guidelines. Our primary goal was to identify areas for improvement in pediatricians’ practices using the 2022 AAP recommendations. By sharing these findings, we aim to promote continuous improvements in neonatal care and inform future educational initiatives that support adherence to the 2022 AAP guidelines.

## 2. Materials and Methods

The members of the New Jersey Chapter of the American Academy of Pediatrics (AAP), which consists of 1890 members, served as the sample frame for our survey study. The New Jersey AAP distributed the survey questionnaire along with an electronic Informed Consent form that outlined the purpose and structure of the study to pediatricians twice over three weeks in November 2024. To confirm their participation, pediatricians were required to click the “I Agree” button. Due to the arrangement of the New Jersey AAP membership database, we were unable to narrow down the sample to target only pediatricians who provide care for neonates with hyperbilirubinemia. Therefore, the survey included the question “Do you provide care for neonates with hyperbilirubinemia?” Pediatricians who responded ‘Yes’ to this question were included in this study. We also excluded residents, medical students, and retired pediatricians from the study population. Survey responses were collected via the Qualtrics platform, which ensured robust data security and maintained the anonymity of respondents. The study was conducted in accordance with the Declaration of Helsinki and approved by the Institutional Review Board of Rutgers University (Pro2024002413 and 9 December 2024).

### 2.1. Survey Instrument

We developed the survey questionnaire based on key practical points from the 2022 guidelines (File S1). This approach ensured that the responses genuinely reflected the knowledge necessary for effective management of neonatal hyperbilirubinemia. To enhance clarity and usability, we pretested the survey instrument with two pediatric hospitalists, allowing us to identify areas for improvement. Based on their feedback, we made several adjustments to simplify the questionnaire, and the study team subsequently approved the final version. This collaborative process strengthened the quality and effectiveness of our tool. The survey consisted of 28 closed-ended questions designed to evaluate pediatricians’ knowledge and practices concerning the management of neonatal hyperbilirubinemia, in accordance with the 2022 AAP guidelines. In addition, demographic questions related to pediatric specialties, practice types, gender, and years of experience were evaluated.

The main topics of these questions included (i) identifying the significance of neurotoxicity related to factors associated with hyperbilirubinemia; (ii) compliance with the recommendation to initiate phototherapy at bilirubin levels higher than those previously recommended; (iii) discontinuing phototherapy when bilirubin levels fall below 2 mg/dL at initiation; (iv) determining the necessity for follow-up and repeat bilirubin testing after discharge based on the pre-discharge bilirubin level; (v) adhering to recommendations for additional blood testing; (vi) assessing the difference between the pre-discharge bilirubin level and the hour-specific phototherapy threshold; and (vii) determining the necessity for follow-up and repeat bilirubin testing after discharge based on the pre-discharge bilirubin level.

We utilized a Likert scale to collect participants’ responses to key questions, providing options such as “Always,” “Often,” “Rarely,” or “Never.” Questions left unanswered by pediatricians who responded to the demographic questions were marked as “No answer,” providing insight into participant engagement.

### 2.2. Statistical Analysis

We identified demographic differences between respondents and non-respondents to the survey question. This was done by using cross-tabulation for categorical data (such as gender, practice type, and subspecialty) and a t-test for independent samples for continuous data (such as age). The data is presented as proportions and means, accompanied by 95% confidence intervals (95% CI). We calculated percentages from ordinal data representing Likert-scale responses, as shown in Table 1, Table 2, Table 3 and Table 4. The denominator consisted of 120 responses detailing their involvement in managing infants with hyperbilirubinemia. Statistical analysis was conducted using STATISTICA version 14.01 (TIBCO Software Inc., Palo Alto, CA, USA).

## 3. Results

**Figure 1:**
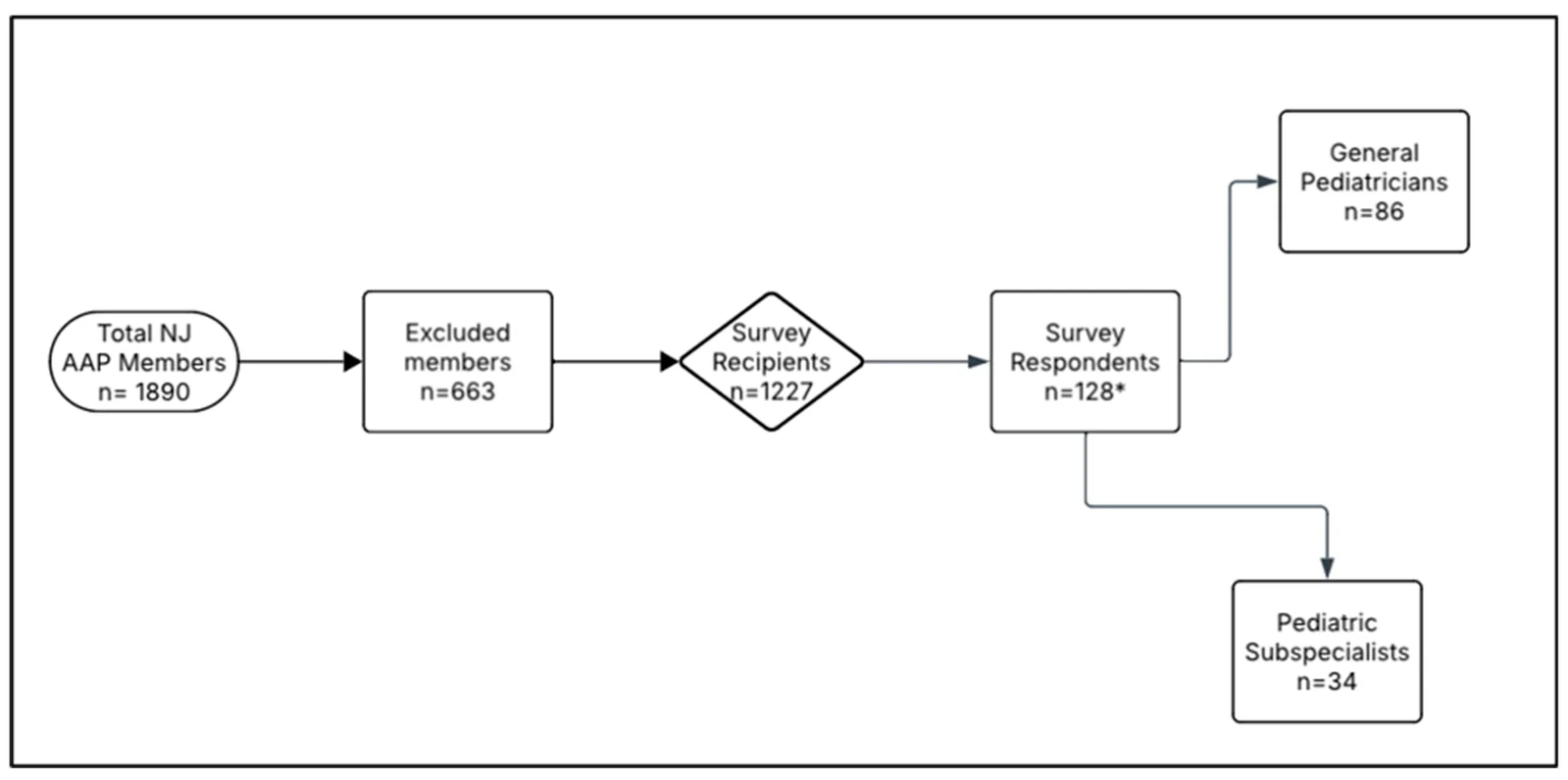
Flow diagram of surveyed pediatricians. * Eight respondents indicated no involvement with newborns and were excluded from the final analysis.

A total of 128 responses were received from a pool of hospital-based (*n* = 610) and private pediatricians (*n* = 625) from the New Jersey Chapter of AAP regarding their management of neonates with hyperbilirubinemia. Out of 128 respondents, eight pediatricians reported no involvement in management of newborns with hyperbilirubinemia and therefore were excluded from the final analysis (Figure 1). Residents, medical students, and retired pediatricians were excluded, resulting in a total of 655 individuals being removed from the denominator. The adjusted response rate of 10.4% (*n* = 120) was calculated from the 1227 eligible respondents. All respondents provided valuable demographic information, detailing their gender, practice type, practice setting, and years of professional experience.

### 3.1. Demographic Characteristics of Study Participants

General pediatricians and pediatric subspecialists comprised 86 (71.7%, 95% CI 62.6%, 79.3%) and 34 (28.3%, 95% CI 22.2%, 39.2%) of the respondents, respectively (*p* < 0.001). Among the 34 pediatric subspecialties, 50% (*n* = 17) were neonatologists, 26.6% (*n* = 9) practiced pediatric hospital medicine, and 23.5% (*n* = 8) were pediatricians working in other areas of pediatrics (*p* < 0.01). The primary practice settings for respondents included hospitals (42.5%, 95% CI 33.7%, 51.4%), pediatric primary care settings (48.3%, 95% CI 39.4%, 57.3%), and a combination of both hospitals and pediatric primary care settings (9.2%, 95% CI 4.0%,14.3%) (*p* < 0.01). Female pediatricians accounted for 70.8% (*n* = 85) of study participants. The duration of practice ranged from 0.5 to 46 years, with a mean of 19.0 years (95% CI 16.9 to 21.2).

Of the 120 respondents, 22 did not respond to any questions about the proposed changes to the 2022 AAP guidelines. Females constituted the majority of pediatricians who responded (69.4%, 95% CI 59.3, 78.3%) and of pediatricians who did not respond to specific questions (86.4%, 95% CI 65.1, 97.1%) (*p* = 0.21). The difference in years of practicing pediatrics between respondents and non-respondents to specific questions did not reach statistical significance (19.9, 95%CI 17.5, 22.3 vs. 15.0%; 95%CI 8.8, 21.2 years), *p* = 0.09. General pediatricians accounted for 81.8% (95% CI 59.7, 94.8) of 22 non-respondents and 69.4% (95% CI 59.3, 78.3%) of 98 respondents to the specific questions (*p* = 0.22).

### 3.2. Pediatricians‘ Consideration of Risk Factors for Severe Hyperbilirubinemia (%)

Nearly 18–19% of pediatricians did not respond to the survey questions. Most pediatricians consistently identified significant risk factors for the development of severe hyperbilirubinemia, such as lower gestational age, levels of TSB and TcB, jaundice within the first 24 h after birth, hemolysis, and the provision of phototherapy before discharge (Table 1). Furthermore, pediatricians frequently recognized other risk factors for hyperbilirubinemia, including scalp hematoma, a family history of phototherapy or exchange transfusions, or blood disorders. Less commonly, pediatricians identified Down syndrome and macrosomia in infants of diabetic mothers as potential risks for severe hyperbilirubinemia.

### 3.3. Bilirubin, Blood Tests, and Phototherapy with Respect to Risk Assessment

The survey revealed a significant discrepancy in pediatricians’ responses to the questions presented in Table 2. A small percentage rarely or never considered risk factors when evaluating the risk of severe jaundice in neonates. In low-risk infants, the majority of respondents always tested TcB and nearly 25% always tested TSB within 24-48 h postpartum or before discharge. Regarding obtaining additional tests such as hemoglobin percentage (Hb%), hematocrit percentage (Hct%), or complete blood count (CBC) in infants on phototherapy, pediatricians expressed variability in their practice. Typically, pediatricians do not initiate phototherapy below the recommended threshold. In high-risk infants, pediatricians always practice TSB testing at 6–12 and 24 h after phototherapy. However, only 60% of pediatricians reported always discontinuing phototherapy once TSB levels decrease by at least 2 mg/dL. Most pediatricians reported a lack of use of home phototherapy, as it is less commonly available in New Jersey for hospital treatment of hyperbilirubinemia. Moreover, from 20% to 30% of pediatricians did not respond.

### 3.4. Factors Influencing Decision for Follow-Up and Bilirubin Testing After Discharge

The majority of respondents consistently defined bilirubin level, neurotoxicity risk factors, and weight loss >10% to guide post-discharge follow-up and bilirubin testing in neonates who received phototherapy (Table 3 and Table 4). Postnatal age and breastfeeding were either always or often identified by pediatricians for post-discharge follow-up and bilirubin testing after phototherapy. Only a few respondent pediatricians did not consider the bilirubin threshold, neurotoxicity risk factors, breastfeeding, postnatal age, and weight loss >10% for post-discharge follow-up or bilirubin testing of neonates discharged home. Unfortunately, 18% to 20% of pediatricians did not respond to questions about risk factors used to determine post-discharge follow-up for infants.

## 4. Discussion

In the present study, we employed a survey designed to evaluate pediatricians’ knowledge and practices regarding the revised 2022 AAP recommendations for managing hyperbilirubinemia in neonates born at a gestational age of 35 weeks or greater. The main purpose of this survey study was to highlight existing gaps in knowledge and practice patterns that could guide future educational initiatives. The survey results showed that a significant number of survey participants expressed a strong intention to follow some, but not all, of the recommendations outlined in the revised guidelines. The majority of respondents underscored the necessity of considering neurotoxic risk factors, used risk-based stratification in clinical decision-making for the initiation of phototherapy, and followed the recommended timing for follow-up of bilirubin. Fewer than 50% of surveyed pediatricians recognized Down Syndrome as a risk factor for severe hyperbilirubinemia, though it is well studied that neonates with Down Syndrome have a significantly elevated risk of early hyperbilirubinemia [14,15]. The lack of recognition of Down Syndrome as a risk factor for hyperbilirubinemia by the surveyed pediatricians is unknown. It is possible that this may be due to lack of clinician exposure as birth rates have decreased [14].

With regard to additional laboratory testing for neonates prior to initiating phototherapy and prior to discharge, the pediatricians surveyed expressed varying practices. This could reflect the revised guidelines emphasizing a more individualized approach to laboratory testing for neonates with clinical features concerning for hemolysis rather than for all patients. Substantial reductions in unnecessary laboratory testing while maintaining patient safety have been demonstrated with the implementation of the AAP recommendations for laboratory testing, which invariably has contributed to reduced healthcare costs [9,12,16]. Sukkar et al. reduced the monthly TSB testing rate from 51 to 26.3 tests per 100 patient-days, a 48% reduction by implementing a multidisciplinary intervention that included faculty buy-in, redesigning the hyperbilirubinemia pathway, and incorporating bilirubin risk assessment via smart phrases in the electronic health record [17]. The smart phrases in this study were used to standardize communication with the medical team and nursing, provided a step-by-step approach for interns to delineate the most recent TcB or TSB levels and categorize those levels based on their risk zone [17]. This reduction in testing was achieved with no change in predischarge bilirubin screening rates, length of stay, phototherapy rates, or readmission rates, demonstrating that selective testing does not compromise clinical outcomes.

In our study, only a few pediatricians indicated initiating phototherapy at a lower threshold than the 2022 AAP guidelines recommend, which is now 1-2 mg/dL higher than before. High adherence to the new guidelines represents a significant achievement, particularly as many pediatricians surveyed before the release of the 2022 AAP guidelines were concerned about the change in the bilirubin threshold for phototherapy initiation [12]. Importantly, the history of phototherapy practices shows a general tendency for the initiation of phototherapy at lower TSB levels. In our previous survey of the New Jersey AAP members conducted in 2003-2004, nearly 60% and 30% of pediatricians reported initiation of phototherapy at TSB levels lower than the 1994 AAP guidelines recommended if the infant was more or less than 72 h old, respectively [18]. Moreover, the survey conducted in 1992 recorded the initiation of phototherapy at lower TSB levels by neonatologists [19]. The recorded compliance with the 2022 AAP guidelines for initiating phototherapy highlights pediatricians’ commitment to best practices and the provision of quality neonatal care by reducing unnecessary exposure to phototherapy. A prior quality improvement study revealed a significant decrease in use of phototherapy but a longer duration of phototherapy [9].

Our survey revealed that a limited number of pediatricians consistently followed 2022 AAP recommendations for phototherapy discontinuation when bilirubin levels drop by 2 mg/dL below the initial threshold, though the risk for rebound is relatively small and primarily associated with evidence of hemolysis [20,21]. In accordance with findings in our survey, a retrospective observational study from Israel revealed a significant level of non-adherence to the recommended 2022 AAP guidelines parameters for discontinuation of phototherapy [22]. Nevertheless, participants in the BORN Study reported discontinuation of phototherapy at a threshold of 2 mg/dL lower than the phototherapy initiation in 76% of 67 US hospitals irrespective of the results from the direct antiglobulin test [13]. To our knowledge there is only one experimental study that showed rebound in 22% and 7% of neonates after discontinuing phototherapy below the treatment threshold at lower margin (3 mg/dL) and greater margin (6 mg/dL), respectively [23]. The risk for rebound could be dependent on threshold of bilirubin discontinuation; however, more research is needed since the recommendation for phototherapy discontinuation at 2 mg/dL relies on expert opinion and consensus rather than solid evidence. There may be a tendency for some pediatricians to continue phototherapy beyond the recommended early discontinuation potentially due to concerns for lack of timely follow-up especially if infant is being discharged on a weekend, given the lower risks associated with phototherapy [24]. However, etiologies for prolonged phototherapy usage were not assessed in this study. Though not analyzed in this study, prolonged phototherapy may potentially contribute to increased hospital length of stay and increased health care related costs. As discussed in the 2022 AAP guidelines consequences of phototherapy include infant–family separation, parental anxiety, and disruption of the breastfeeding relationship [8].

In our study, we found that the demographic characteristics and practice types of 22 pediatricians who did not respond to any questions about managing hyperbilirubinemia were similar to those of the pediatricians who did respond. Furthermore, these pediatricians indicated that they were familiar with the 2022 AAP guidelines and actively involved in the management of neonatal hyperbilirubinemia. The lack of responses to the practical questions may stem from a perceived lack of applicability to their daily work or from the absence of definitive scientific answers in the medical literature [25]. It is important to emphasize that knowing clinical practice guidelines does not always ensure their implementation in everyday practice, especially in healthcare settings that may encounter various challenges [26].

This study recognizes certain limitations that warrant attention. First, the response rate, which is below the average of 29% among pediatricians [27], raises concerns about the potential for nonresponse bias, recall bias, self-report bias, and social desirability bias. As a result of the low response rate, the findings may not be generalizable to all New Jersey pediatricians or pediatricians nationally. Nonetheless, it is important to acknowledge that achieving a high response rate in surveys administered to physicians presents significant challenges [28]. Second, the survey was distributed to all members of NJ AAP rather than to the target population of general pediatricians, neonatologists, and hospitalists who are most likely caring for neonates with hyperbilirubinemia. However, to improve the accuracy and efficiency of our study findings, responses from pediatricians who reported their involvement in the clinical management of neonates with hyperbilirubinemia and familiarity with the 2022 AAP guidelines were included in the final analysis, emphasizing the relevance of the data to those directly involved in care. Third, we were only able to conduct a subgroup analysis of demographic data; we did not analyze pediatricians’ responses by specialty or conduct subgroup analyses. This may have further highlighted specific differences in management among general pediatricians, hospitalists, and neonatologists [25]. Furthermore, the survey that was utilized was not a validated survey, which may have impacted the responses.

## 5. Conclusions

Respondents reported practices generally consistent with the 2022 AAP guidelines on key risk factors for hyperbilirubinemia and the bilirubin level at which phototherapy is initiated. However, variability in practice management remains regarding essential recommendations for laboratory testing of patients on phototherapy, as well as for phototherapy discontinuation.

## Figures and Tables

**Table 1:** The discrepancies in pediatricians’ responses regarding the recognition of risk factors for development of severe hyperbilirubinemia (%).

Risk Factors	Always (%)	Often (%)	Rarely (%)	Never (%)	No Answer (%)
Lower GA	70.0	10.0	1.7	0	18.3
TSB or TcB levels	68.3	7.5	5.8	0	18.2
Jaundice before 24 h	72.5	5.8	2.5	0	19.2
Hemolysis	65.0	13.3	3.3	0	18.3
Phototherapy prior to discharge	61.7	16.7	1.7	0.8	19.2
Scalp hematoma	49.2	26.7	5.8	0	18.3
Down Syndrome	22.5	24.2	30.0	5.0	18.3
Macrosomia	26.7	28.3	24.2	1.7	19.2
Family history of phototherapy or exchange transfusion	40.8	30.8	9.2	0.8	18.3
Family history of blood disorders	35.8	30.8	12.5	1.7	19.2

**Table 2:** Obtaining bilirubin and other tests for phototherapy initiation (%).

Management	Always (%)	Often (%)	Rarely (%)	Never (%)	No Answer (%)
Obtaining TcB at 24–48 h postpartum or before discharge in low-risk infants	59.2	1.7	3.3	5.8	30.0
Obtaining TSB at 24–48 h postpartum or before discharge in low-risk infants	25.8	10.0	32.5	8.3	23.3
Initiate phototherapy below recommended threshold	2.5	10.0	54.2	12.5	20.8
Hb %, Hct%, or CBC testing in all patients on phototherapy	26.7	20.8	28.3	4.2	20.0
Discontinue phototherapy if TSB reduced by at least 2 mg/dL below the hour-specific threshold at which treatment was originally initiated	12.5	47.5	11.7	1.7	26.7
TSB at 6–12 and 24 h after phototherapy in high-risk infants	43.3	26.7	5.8	1.7	22.5
Home phototherapy use	0	3.3	10.0	58.3	28.3
Daily TSB if home phototherapy is provided	10.0	1.7	0.8	58.3	29.2

**Table 3:** Factors influencing decision for post-discharge follow-up (%).

Factors	Always (%)	Often (%)	Rarely (%)	Never (%)	No Answer (%)
Bilirubin and phototherapy threshold	52.5	25.8	2.5	0.83	18.3
* Neurotoxicity risk factors	63.3	16.7	0.83	0.83	18.3
Postnatal age	43.3	30.8	5.0	1.7	19.2
Breastfeeding	46.7	30.8	3.3	0.83	18.3
Weight loss > 10%	58.3	18.3	4.2	0.83	18.3

* Gestational age < 38 weeks, albumin < 3.0 g/dL, isoimmune hemolytic disease, sepsis, or clinical instability in the previous 24 h.

**Table 4:** Factors influencing decision for post-discharge bilirubin testing (%).

Factors	Always (%)	Often (%)	Rarely (%)	Never (%)	No Answer (%)
Bilirubin after phototherapy	53.3	23.3	4.2	0.83	18.3
* Neurotoxicity risk factors	58.3	21.7	0	0	20.0
Postnatal age	38.3	34.2	7.5	1.7	18.3
Breastfeeding	35.0	35.0	8.3	1.7	19.2
Weight loss > 10%	45.0	25.8	9.2	0.83	19.2

* Gestational age < 38 weeks, albumin < 3.0 g/dL, isoimmune hemolytic disease, sepsis, or clinical instability in the previous 24 h.

## Data Availability

The original contributions presented in this study are included in the article. Further inquiries can be directed to the corresponding author.

## Supplementary Materials

The following supporting information can be downloaded at: https://www.mdpi.com/article/10.3390/pediatric18040101/s1, File S1: Pediatrician’s Knowledge of the AAP 2022 Revised Hyperbilirubinemia Management Guidelines Survey Questions.

## Abbreviations

The following abbreviations are used in this manuscript:

AAP	American Academy of Pediatrics
TcB	Transcutaneous Bilirubin
TSB	Total Serum Bilirubin
DAT	Direct antiglobulin test
