# Supplementary material for: Pediatricians’ Practice Patterns on the Revised 2022 Neonatal Hyperbilirubinemia Guidelines
Source: Pediatr Rep. 2026 Aug 3;18(4):101. doi: 10.3390/pediatric18040101 (PMC13516341; doi:10.3390/pediatric18040101)
Supplement: Supplementary file 1 [file pediatrrep-18-00101-s001.zip › pediatrrep-4344156-supplementary.pdf]

**Pediatrician's Knowledge of the AAP 2022 Revised Hyperbilirubinemia Management Guidelines Survey Questions**

Demographic Questions

- Do you provide care to newborns with hyperbilirubinemia?
    - Yes
    - No
- Are you familiar with the revised hyperbilirubinemia guidelines?
    - Yes
    - No
- What is your specialty? 1) General Pediatrician 2)Subspecialty, please specify
- Which of the following describes your practice? (Check all that apply)
    - University/Community based hospital
    - Pediatric practice
    - Other, please specify
- How many years have you been in pediatric practice?
- With which gender do you most identify?
    - Male
    - Female
    - Other, please specify
    - Prefer not to answer

Practice Management Questions

When managing infants 35 or more weeks gestation, do you consider the following risk factors for development of severe hyperbilirubinemia?

| | Always | Often | Rarely | Never |
|---|---|---|---|---|
| Lower gestational age | | | | |
| Jaundice first 24 hours after birth | | | | |
| Serum or Transcutaneous Bilirubin prior to discharge | | | | |
| Hemolysis | | | | |
| Phototherapy prior to discharge | | | | |
| Family history of phototherapy or exchange transfusion | | | | |
| Family history of red blood cell disorders | | | | |
| Scalp hematoma or significant bruising | | | | |
| Down syndrome | | | | |
| Macrosomic infant of a diabetic mother | | | | |

Please answer the following questions regarding your management of hyperbilirubinemia in infants 35 or more weeks gestation.

| | Always | Often | Rarely | Never |
|---|---|---|---|---|
| Obtain TRANSCUTANEOUS bilirubin between 24 and 48 hours after birth or before discharge in low risk infants? | ○ | ○ | ○ | ○ |
| Obtain SERUM bilirubin between 24 and 48 hours after birth or before discharge in low risk infants? | ○ | ○ | ○ | ○ |
| Initiate phototherapy below recommended phototherapy threshold? | ○ | ○ | ○ | ○ |
| Obtain hemoglobin, hematocrit or complete blood count in all patients requiring phototherapy | ○ | ○ | ○ | ○ |
| Discontinue phototherapy for patients who have serum bilirubin reduction by at least 2 mg/dL? | ○ | ○ | ○ | ○ |
| Obtain serum bilirubin 6 -12 hours and 24 hours after discontinuation of phototherapy in high risk infants [received phototherapy before 48 hours of age, had a positive DAT or who have known or suspected hemolytic disease] | ○ | ○ | ○ | ○ |
| Provide home phototherapy | ○ | ○ | ○ | ○ |
| If home phototherapy provided do you obtain daily serum bilirubin? | ○ | ○ | ○ | ○ |

Do the following factors influence your decision when determining timing of post discharge follow up?

| | Always | Often | Rarely | Never |
|---|---|---|---|---|
| Difference between discharge bilirubin and phototherapy therapy threshold | ○ | ○ | ○ | ○ |
| Hyperbilirubinemia Neurotoxicity risk factors [<38 gestational age, Albumin <3.0 g/dL, hemolytic disease, sepsis, clinical instability in previous 24hrs)] | ○ | ○ | ○ | ○ |
| Postnatal age | ○ | ○ | ○ | ○ |
| Assessment of breastfeeding | ○ | ○ | ○ | ○ |
| Weight loss more than 10% | ○ | ○ | ○ | ○ |

Do the following factors influence your decision when obtaining additional transcutaneous or serum bilirubin after discharge?

| | Always | Often | Rarely | Never |
|---|---|---|---|---|
| Difference between discharge bilirubin and phototherapy therapy threshold | | | | |
| Hyperbilirubinemia Neurotoxicity risk factors [<38 gestational age, Albumin <3.0 g/dL, hemolytic disease, sepsis, clinical instability in previous 24hrs)] | | | | |
| Postnatal age | | | | |
| Assessment of breastfeeding | | | | |
| Weight loss more than 10% | | | | |
